# Association between glymphatic dysfunction and glucose hypometabolism in chronic disorders of consciousness: a multimodal PET/MRI study

**DOI:** 10.1093/braincomms/fcag235

**Published:** 2026-06-24

**Authors:** Yirong Wang, Kun Guo, Qi Zhang, Zhiyong Quan, Guiyu Li, Taoqi Ma, Yifei Zhang, Jiehui Jiang, Jing Wang, Fei Kang

**Affiliations:** Department of Nuclear Medicine, Xijing Hospital, Fourth Military Medical University, Xi’an, Shaanxi 710032, China; Department of Nuclear Medicine, Xijing Hospital, Fourth Military Medical University, Xi’an, Shaanxi 710032, China; School of Communication & Information Engineering, Shanghai University, Shanghai 200444, China; Department of Nuclear Medicine, Xijing Hospital, Fourth Military Medical University, Xi’an, Shaanxi 710032, China; Department of Nuclear Medicine, Xijing Hospital, Fourth Military Medical University, Xi’an, Shaanxi 710032, China; Department of Nuclear Medicine, Xijing Hospital, Fourth Military Medical University, Xi’an, Shaanxi 710032, China; Department of Diagnostic and Interventional Neuroradiology, Klinikum Rechts der Isar, Technical University of Munich, Munich 81675, Germany; School of Life Sciences, Shanghai University, Shanghai 200444, China; Department of Nuclear Medicine, Xijing Hospital, Fourth Military Medical University, Xi’an, Shaanxi 710032, China; Department of Nuclear Medicine, Xijing Hospital, Fourth Military Medical University, Xi’an, Shaanxi 710032, China

**Keywords:** disorders of consciousness, PET/MRI, glymphatic system, glucose metabolism

## Abstract

The glymphatic system is essential for removing metabolic waste from the brain and facilitating the distribution of nutrients, but its status in patients with chronic disorders of consciousness (DoC) remains poorly understood. This study evaluated glymphatic function in chronic DoC and investigated its association with regional cerebral glucose metabolism. Forty-one patients with chronic DoC and 26 healthy controls (HCs) were retrospectively included. All participants underwent ^18^F-fluorodeoxyglucose (^18^F-FDG) positron emission tomography/magnetic resonance imaging (PET/MRI). Glymphatic function was quantified using the diffusion tensor image analysis along the perivascular space (DTI-ALPS) index. Standardized uptake value ratios (SUVRs) of key hubs within the ‘mesocircuit model’ were calculated using the cerebellum as reference. The characteristic glucose metabolic pattern (GMP) of chronic DoC was identified, and its expression scores were computed. Associations between the glymphatic function and cerebral glucose metabolism, behavioural score and serological markers were evaluated. Patients with chronic DoC showed significantly lower DTI-ALPS indices than HCs (*P* < 0.001). Within the patient group, moderate to strong correlations were observed between the DTI-ALPS index and the SUVRs of mesocircuit-associated regions, including the frontal cortex (*r* = 0.335, *P* = 0.002), parietal cortex (*r* = 0.383, *P* < 0.001), thalamus (*r* = 0.528, *P* < 0.001), brainstem (*r* = 0.665, *P* < 0.001), striatum (*r* = 0.537, *P* < 0.001) and globus pallidus (*r* = 0.372, *P* = 0.001), but not with the GMP score, behavioural score, or serological markers (all *P* > 0.05). Multivariate analysis further revealed that older age (*β* = −0.312, *P* < 0.001) and lower SUVRs in the parietal cortex (*β* = 0.196, *P* = 0.030) and brainstem (*β* = 0.482, *P* < 0.001) independently predicted reduced DTI-ALPS indices. This study provides *in vivo* evidence that glymphatic dysfunction is significantly associated with regional cerebral hypometabolism in chronic DoC, particularly in the parietal cortex and brainstem, which may serve as physiological drivers of CSF circulation.

## Teaser

Wang *et al.* report that patients with chronic disorders of consciousness exhibit impaired brain fluid clearance linked to reduced glucose metabolism, particularly in the parietal cortex and brainstem. These findings suggest that regional brain activity may help drive cerebrospinal fluid circulation and highlight a novel mechanism underlying disorders of consciousness.

## Graphical Abstract

**Graphical Abstract:**
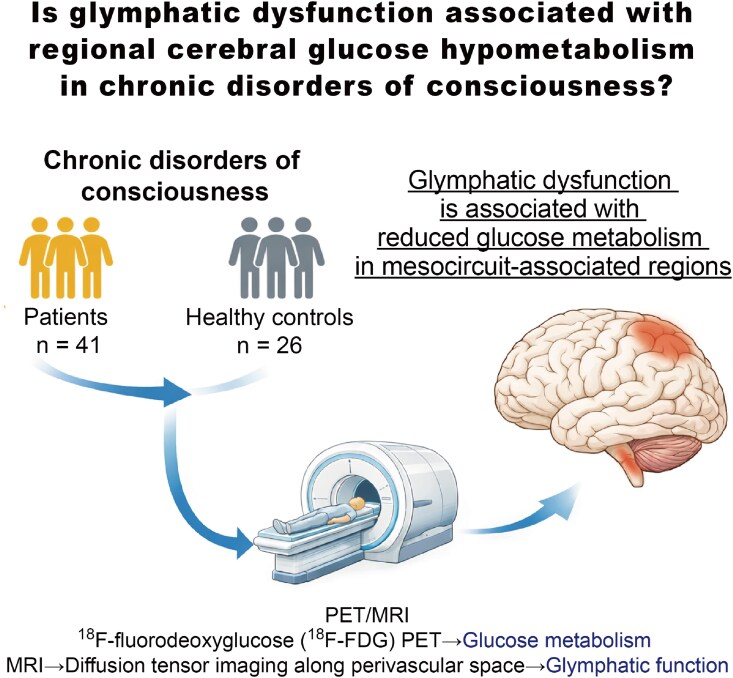

## Introduction

Chronic disorders of consciousness (DoC) primarily include unresponsive wakefulness syndrome (UWS) and the minimally conscious state (MCS).^1,2^ Patients with chronic DoC present with absent or minimal clinically detectable awareness, imposing substantial long-term care burdens on healthcare systems and families.^3^ Despite advances in neurological management, many patients fail to fully regain awareness, highlighting critical gaps in understanding the pathophysiological mechanisms underlying sustained unconsciousness.^4^ Identifying reliable, quantifiable biomarkers that capture these mechanisms is therefore essential for early diagnosis, prognostic evaluation and the development of targeted interventions.

Cerebral glucose metabolism, measured via ^18^F-fluorodeoxyglucose positron emission tomography (^18^F-FDG PET), is a well-established biomarker for chronic DoC.^5^ Extensive studies have shown a global reduction in glucose utilization, particularly within key nodes of the consciousness network such as the frontoparietal cortex.^6,7^ This widespread cerebral hypometabolism is strongly correlated with the severity and chronicity of impaired consciousness, reflecting both a consequence of reduced neuronal activity and a barrier to large-scale neural network reintegration necessary for awareness.^8^

Recent research has highlighted the essential function of brain waste clearance in preserving neural balance. The glymphatic system, which operates through cerebrospinal fluid (CSF) inflow along the perivascular space and is mediated by astrocytic aquaporin-4 water channels, effectively eliminates metabolic by-products, including neurotoxic substances such as β-amyloid and tau proteins.^9,10^ Disruption of glymphatic clearance has been linked to the development of multiple neurodegenerative diseases and is thought to contribute to secondary neuronal damage.^11,12^ Growing evidence also indicates that brain injury can impair glymphatic circulation through neuroinflammation and astrocytic dysfunction, thereby reducing cerebrospinal fluid movement and waste clearance.^9,13^ In patients with chronic DoC, such disruption may facilitate the accumulation of neurotoxic metabolites and contribute to persistent neural dysfunction.^14^

Emerging evidence suggests a bidirectional interaction between glymphatic flow and neural metabolism. Neural oscillations are thought to play an important role in driving glymphatic perfusion.^15^ Reduced neuronal activity may therefore weaken these forces, impairing CSF movement. Conversely, impaired glymphatic clearance could exacerbate neuronal dysfunction via waste accumulation, forming a pathological feedback loop. Despite its potential importance, this bidirectional interaction has not been systematically investigated in chronic DoC. The diffusion tensor image analysis along the perivascular space (DTI-ALPS) index, a noninvasive MRI-based measure, allows quantification of glymphatic diffusivity by assessing water movement along periventricular perivascular space.^16^ Alterations in DTI-ALPS have been observed in conditions like Alzheimer’s and frontotemporal dementia,^17-19^ but whether similar changes exist in chronic DoC and how they relate to brain metabolism is unknown.

In this study, we aimed to (1) compare glymphatic function between patients with chronic DoC and healthy controls (HCs) via the DTI-ALPS index and (2) investigate the associations between the glymphatic function and cerebral glucose metabolism in patients with chronic DoC.

## Materials and methods

### Participants

Patients with chronic DoC who underwent ^18^F-FDG PET/MRI at Xijing Hospital between August 2022 and December 2023 were retrospectively enrolled. The level of consciousness was evaluated using the Coma Recovery Scale-Revised (CRS-R) in combination with ^18^F-FDG PET imaging. An experienced neuropsychologist conducted CRS-R assessments daily for five consecutive days, and the highest score obtained during this period was recorded. If the results were ambiguous, two nuclear medicine physicians visually reviewed the frontoparietal cortex glucose metabolism on ^18^F-FDG PET. UWS was diagnosed when the associative frontoparietal cortex showed complete bilateral hypometabolism, whereas MCS was determined by the presence of residual metabolic activity within these regions. Any differences were adjudicated by a third physician if necessary. In cases where team consensus could not be reached, patients were reassessed until agreement was achieved. Serum neuron-specific enolase (NSE) and S100 calcium-binding protein B (S100B) levels measured within ten days of the PET/MRI scan were retrieved from electronic medical records, and all assays were conducted following standardized protocols. Biomarker measurements obtained outside this predefined time window were not included in the analysis.

The inclusion criteria were: (i) level of consciousness established by the aforementioned comprehensive assessment, (ii) at least 28 days had elapsed since brain injury and (iii) older than 18 years. The exclusion criteria were: (i) extensive focal brain lesions involving more than two-thirds of a hemisphere or significant brain distortion due to surgery or trauma as determined by a board-certified neuroradiologist, and (ii) inadequate image quality, including excessive motion artefacts, significant signal dropout, failed spatial registration, or inability to reliably place periventricular regions of interest (ROIs). The patient selection process is summarized in Supplementary Fig. 1.

Additionally, 26 healthy controls, matched by age and sex and confirmed to have no history of psychiatric or neurological illness through health screening, were included.

This study was performed in line with the principles of the Declaration of Helsinki and was approved by the Ethics Committee of Xijing Hospital (No. KY20243056-1). Because this study was conducted retrospectively, the need for written informed consent was waived.

### PET/MRI imaging procedure

All patients with chronic DoC and HCs underwent ^18^F-FDG PET/MR imaging (SIGNA PET/MR; GE Healthcare, WI, USA). All participants were required to fast for at least six hours to ensure that their blood glucose levels remained below 11.1 mmol/l, and then 3.7 MBq/kg ^18^F-FDG was injected intravenously. After resting in a quiet environment for 40 min, PET and MRI data were collected concurrently using a vendor-provided 8-channel head coil. No patient received sedatives or anaesthetics to avoid motion artefacts during scanning.

The MRI protocol included several sequences. A 3D T_1_-weighted sagittal scan was acquired with the following parameters: TR = 6.9 ms, TE = 2.98 ms, inversion time = 450 ms, flip angle = 12°, matrix = 256 × 256, FOV = 256 × 256 mm^2^, slice thickness = 1 mm, 192 continuous sagittal slices and an isotropic voxel size of 1 × 1 × 1 mm^3^. DTI data were acquired using a spin-echo echo-planar imaging sequence, with five volumes at *b* = 0 s/mm^2^ and 32 diffusion-weighted directions at *b* = 1000 s/mm^2^. DTI settings included TR = 16 500 ms, TE = 74.6 ms, matrix = 112 × 112, FOV = 256 × 256 mm^2^, 70 axial slices at 2 mm thickness without gaps and one excitation. PET images were collected as static acquisitions with a matrix of 192 × 192, FOV of 350 × 350 mm^2^ and voxel dimensions of 1.82 × 1.82 × 2.78 mm^3^. All PET data were corrected for photon attenuation, random events, scatter and system dead time. Foam cushions were placed within the head coil to reduce head motion during the scan.

### Quantification of the DTI-ALPS index

**Figure 1:**
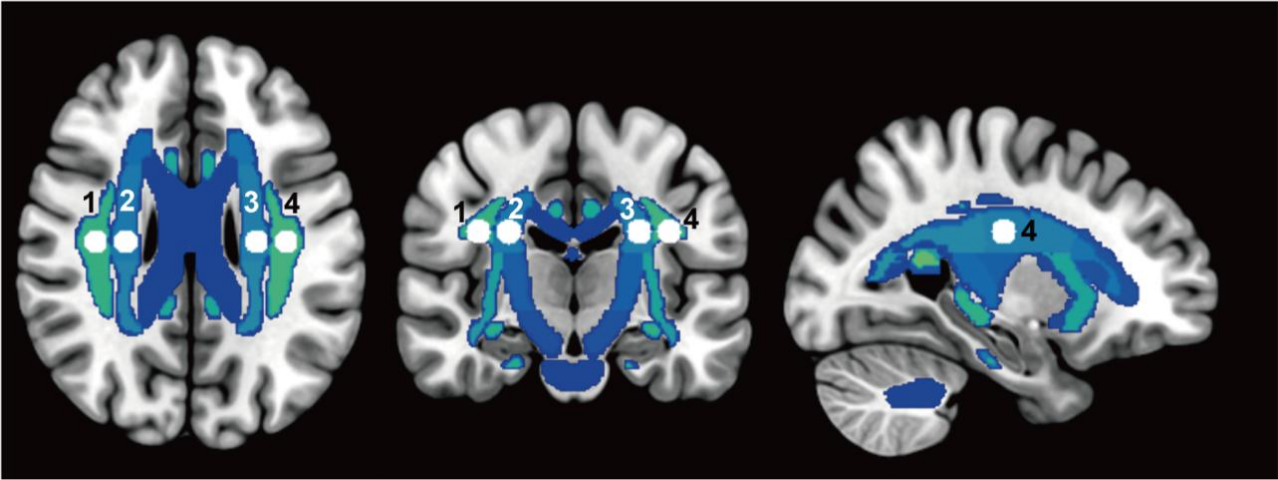
Schematic of the MRI processing approach for DTI-ALPS calculation. White dots indicate the ROIs used for diffusivity measurement. The numbers denote the corresponding white matter tracts: 1 = Association fibre, right superior longitudinal fasciculus (SLF); 2 = Projection fibre, right superior corona radiata (SCR); 3 = Projection fibre, left superior corona radiata (SCR); 4 = Association fibre, left superior longitudinal fasciculus (SLF). DTI-ALPS, diffusion tensor image analysis along the perivascular space; ROI, region of interest.

DTI data were processed to compute the DTI-ALPS index.^20^ First, raw diffusion-weighted images in DICOM format were converted into NIfTI format using the MRIcroGL graphical interface (MRIcroGL, McCausland Center for Brain Imaging, University of South Carolina, Columbia, SC, USA). Thermal noise was removed using the Marchenko-Pastur principal component analysis denoising algorithm via the dwidenoise function of MRtrix3 (MRtrix3, Brain Research Institute, Melbourne, VIC, Australia). Thereafter, Gibbs ringing artefacts were corrected using the mrdegibbs function of MRtrix3. Eddy current and head motion corrections were performed using the eddy tool in FSL (FMRIB Software Library version 6.0, Oxford Centre for Functional MRI of the Brain, Oxford University, Oxford, Oxfordshire, UK). Head motion was evaluated using displacement estimates and slice outlier detection generated by the FSL eddy preprocessing pipeline. Datasets showing excessive motion artefacts, signal dropout, or unsuccessful correction were excluded after quality control. After artefact correction, diffusion tensors were fitted using the dtifit function in FSL to generate fractional anisotropy (FA) maps and directional diffusivity maps along the *x*-axis (right-left; Dxx), *y*-axis (anterior-posterior; Dyy) and *z*-axis (inferior-superior; Dzz). The fractional anisotropy map of each subject was first linearly registered to the JHU-ICBM-fractional anisotropy template (defined in MNI152 space) using FSL’s flirt function, followed by nonlinear registration with fnirt. Registration accuracy was visually inspected for each subject, and datasets with failed registration were excluded. Next, the resulting transformation matrices were applied to the diffusivity maps to bring all subjects into a common template space. ROIs were defined based on the JHU-ICBM-DTI-81 white matter atlas. Projection fibre ROIs were placed in the bilateral superior corona radiata (SCR), and association fibre ROIs were placed in the bilateral superior longitudinal fasciculus (SLF), both at the level of the lateral ventricle body. Spherical ROIs with 5 mm diameter were constructed using the following template-space coordinates: left SCR (11 611 099), right SCR (6 411 099), left SLF (12 811 099) and right SLF (5 111 099). Directional diffusivity values (Dxx, Dyy, Dzz) were extracted from these ROIs (Fig. 1).

The DTI-ALPS index was determined by dividing the mean of the *x*-axis diffusivity in projection fibre regions (Dxxproj) and association fibre regions (Dxxassoc) by the mean of the *y*-axis diffusivity in projection fibre regions (Dyyproj) and the *z*-axis diffusivity in association fibre regions (Dzzassoc), as shown below^16^:


DTI-ALPS=mean(Dxxproj,Dxxassoc)/mean(Dyyproj,Dzzassoc)

The average of the bilateral DTI-ALPS indices was calculated as the whole-brain DTI-ALPS index. To ensure the validity of DTI-ALPS measurements, ROI placement was additionally checked to confirm that regions were located within periventricular white matter and not within cerebrospinal fluid spaces or lesion cavities. Datasets in which reliable periventricular ROI placement could not be achieved were excluded.

### PET image processing

Statistical Parametric Mapping version 12 (SPM12, Wellcome Centre for Human Neuroimaging, University College London, London, UK), implemented in MATLAB R2018b (MathWorks Inc., Natick, MA, USA), was employed to preprocess PET and structural MRI images. T_1_-weighted structural images were first segmented and spatially normalized to the standard MNI152 space using diffeomorphic anatomical registration through the exponentiated Lie algebra framework. The deformation fields generated from T_1_-weighted MRI were then applied to the co-registered PET images to achieve spatial normalization, ensuring anatomical alignment between PET and MRI modalities. To enhance image quality, the normalized PET images were spatially smoothed with a Gaussian kernel of 8 mm full width at half maximum.

The ROI for the brainstem was manually delineated by a neuroimaging specialist and subsequently reviewed and confirmed by a senior neuroimaging specialist with more than 10 years of experience. Delineation was guided by anatomical landmarks, including the contour of the brainstem and the fourth ventricle, to ensure consistent placement across subjects. Other ROIs relevant to the ‘mesocircuit model’ were defined based on the Automated Anatomical Labelling atlas. To standardize regional glucose metabolism, standardized uptake value ratios (SUVRs) were computed with the cerebellum serving as the reference region.

Employing the ScAnVP version 7.0w software package (Feinstein Institutes for Medical Research, Manhasset, NY, USA) running in MATLAB R2018b (MathWorks Inc., Natick, MA, USA), metabolic pattern analysis was conducted using the scaled subprofile model and principal component analysis (SSM/PCA). A binary brain mask was constructed by applying a 35% threshold of the whole-brain intensity maximum to exclude nonbrain voxels. Voxelwise glucose uptake values were then log-transformed across all subjects. PCA was conducted to extract orthogonal principal components characterizing intersubject covariance patterns. Meaningful components were selected based on two criteria: (i) cumulative variance explained > 50% and (ii) inter-group differences in expression scores reaching *P* < 0.2 in two-sample *t*-tests. Selected components were used in a logistic regression model to generate composite spatial patterns and individual expression scores. In the final expression map, voxels with |*z*| ≥ 1.65 (*P* ≤ 0.05) and a cluster size > 100 voxels were considered significant. Subject-specific pattern expression scores were calculated using the topographic profile rating algorithm. These procedures were applied to both patients with chronic DoC and HCs to obtain individual glucose metabolic pattern (GMP) scores for statistical analysis.

### Statistical analysis

All statistical analyses were conducted using SPSS version 23.0 (IBM Corp., Armonk, NY, USA). Two-sample *t*-test or Mann–Whitney U-test was used to compare continuous variables between groups, and the χ^2^ test was used to compare categorical variables between groups. Partial Pearson correlation analysis was conducted to explore the correlations between the DTI-ALPS index and glucose metabolic parameters, CRS-R scores and serological biomarker levels. For correlations involving mesocircuit-associated regions, the false discovery rate (FDR) method was applied to correct for multiple comparisons. Subsequently, using a backward elimination approach, multivariate linear regression analysis was conducted to identify the independent predictors of the DTI-ALPS index in patients with chronic DoC. A *P*-value of < 0.05 was considered statistically significant.

## Results

### Demographic characteristics and clinical features

Table 1 presents the demographic and clinical information for both chronic DoC patients and HCs. Among the 41 patients, 23 were men (56.1%) and 18 were women (43.9%), with a mean age of 48.39 years. There were no statistically significant differences in age or sex distribution between the chronic DoC group and the HCs (*P* > 0.05). Among patients with chronic DoC, 21 (51.2%) were diagnosed with UWS, 20 (48.8%) were diagnosed with MCS. In three patients, repeated CRS-R assessments did not demonstrate reproducible MCS-specific behaviours. These cases therefore underwent PET-based adjudication, and preserved frontoparietal cortical metabolism on ^18^F-FDG PET supported classification as MCS. All remaining patients were classified solely according to CRS-R criteria. Nine patients (22.0%) had a traumatic aetiology, and 32 (78.0%) had a non-traumatic aetiology. The mean serum levels of NSE and S100B were 19.86 ng/ml and 0.08 μg/l, respectively.

**Table 1:** Demographic and clinical features of patients with chronic DoC and HCs

Characteristic	chronic DoC (*n* = 41)	HC (*n* = 26)	*P*
Age, years, mean ± SD	48.39 ± 14.66	49.27 ± 12.83	0.803
Sex, male, *n* (%)	23 (56.1)	15 (57.7)	0.898
Aetiology, *n* (%)			
Traumatic	9 (22.0)		
Non-traumatic	32 (78.0)		
Event occur to PET/MRI scan, d, median (IQR)	50.0 (30.0–82.0)		
Diagnosis at PET/MRI scan, *n* (%)			
UWS	21 (51.2)		
MCS	20 (48.8)		
CRS-R scores at PET/MRI scan, median (IQR)	7.0 (5.0–8.0)		
Serum NSE, ng/ml, median (IQR)	19.86 (15.89–31.15) (*n* = 36)		
Serum S100B, μg/l, median (IQR)	0.08 (0.05–0.12) (*n* = 34)		

CRS-R, Coma Recovery Scale-Revised; DoC, disorders of consciousness; HC, healthy control; MCS, minimally conscious state; MRI, magnetic resonance imaging; NSE, neuron-specific enolase; PET, positron emission tomography; S100B, S100 calcium-binding protein B; UWS, unresponsive wakefulness syndrome.

### Glymphatic dysfunction in patients with chronic DoC

**Figure 2:**
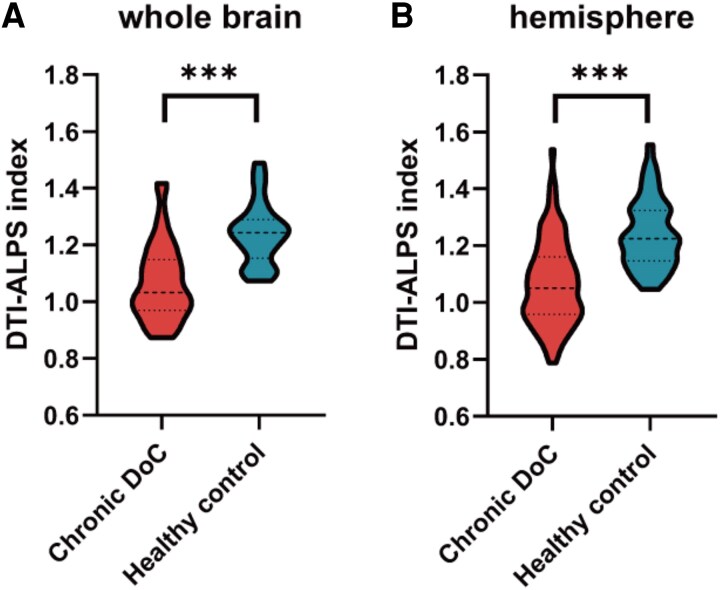
DTI-ALPS index of patients with chronic DoC and healthy controls. Compared with healthy controls (*n* = 26), patients with chronic DoC (*n* = 41) showed significantly lower DTI-ALPS indices at both the whole-brain level (**A**) and hemispheric level (**B**). Group comparisons were performed using the Mann–Whitney U-test (whole brain: U = 164, *P* < 0.001; hemispheric level: U = 768, *P* < 0.001). DoC, disorders of consciousness; DTI-ALPS, diffusion tensor image analysis along the perivascular space.

Patients with chronic DoC showed significantly lower DTI-ALPS indices compared to HCs on both whole-brain and hemispheric levels (both *P* < 0.001) (Fig. 2).

Among HCs, the DTI-ALPS index exhibited a negative correlation with age on whole-brain (*r* = −0.549, *P* = 0.004) and hemispheric (*r* = −0.493, *P* < 0.001) levels. No significant differences were observed between male and female HCs concerning the DTI-ALPS index (both *P* > 0.05 on whole-brain and hemispheric levels).

**Figure 3:**
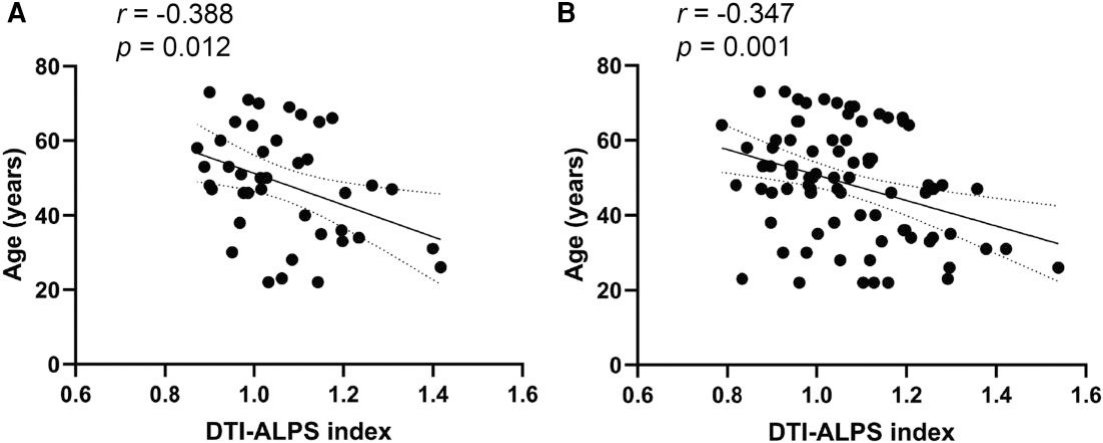
Correlations of the DTI-ALPS index with age in patients with chronic DoC. Pearson correlation analysis was performed to examine the relationship between age and the DTI-ALPS index in patients with chronic DoC (*n* = 41). A negative correlation was observed between age and the DTI-ALPS index at the whole-brain level (**A**) and hemispheric level (**B**). Each dot represents one individual patient. DoC, disorders of consciousness; DTI-ALPS, diffusion tensor image analysis along the perivascular space.

Similarly, in the chronic DoC group, the DTI-ALPS index was negatively correlated with age on whole-brain (*r* = −0.388, *P* = 0.012) and hemispheric (*r* = −0.347, *P* = 0.001) levels (Fig. 3). Concerning the DTI-ALPS index, no significant differences were observed between male and female patients, between those diagnosed with UWS and MCS, or between patients with traumatic aetiologies and those with non-traumatic aetiologies (all *P* > 0.05 on whole-brain and hemispheric levels). In addition, no significant association was observed between the DTI-ALPS index and the interval from event occurrence to PET/MRI scan in patients with chronic DoC at either the whole-brain or hemispheric level (both *P* > 0.05). After excluding the three PET-adjudicated MCS patients, the group comparison between UWS and MCS remained unchanged, confirming the stability of the findings.

### Correlation between glymphatic dysfunction and glucose hypometabolism in mesocircuit-associated regions

**Figure 4:**
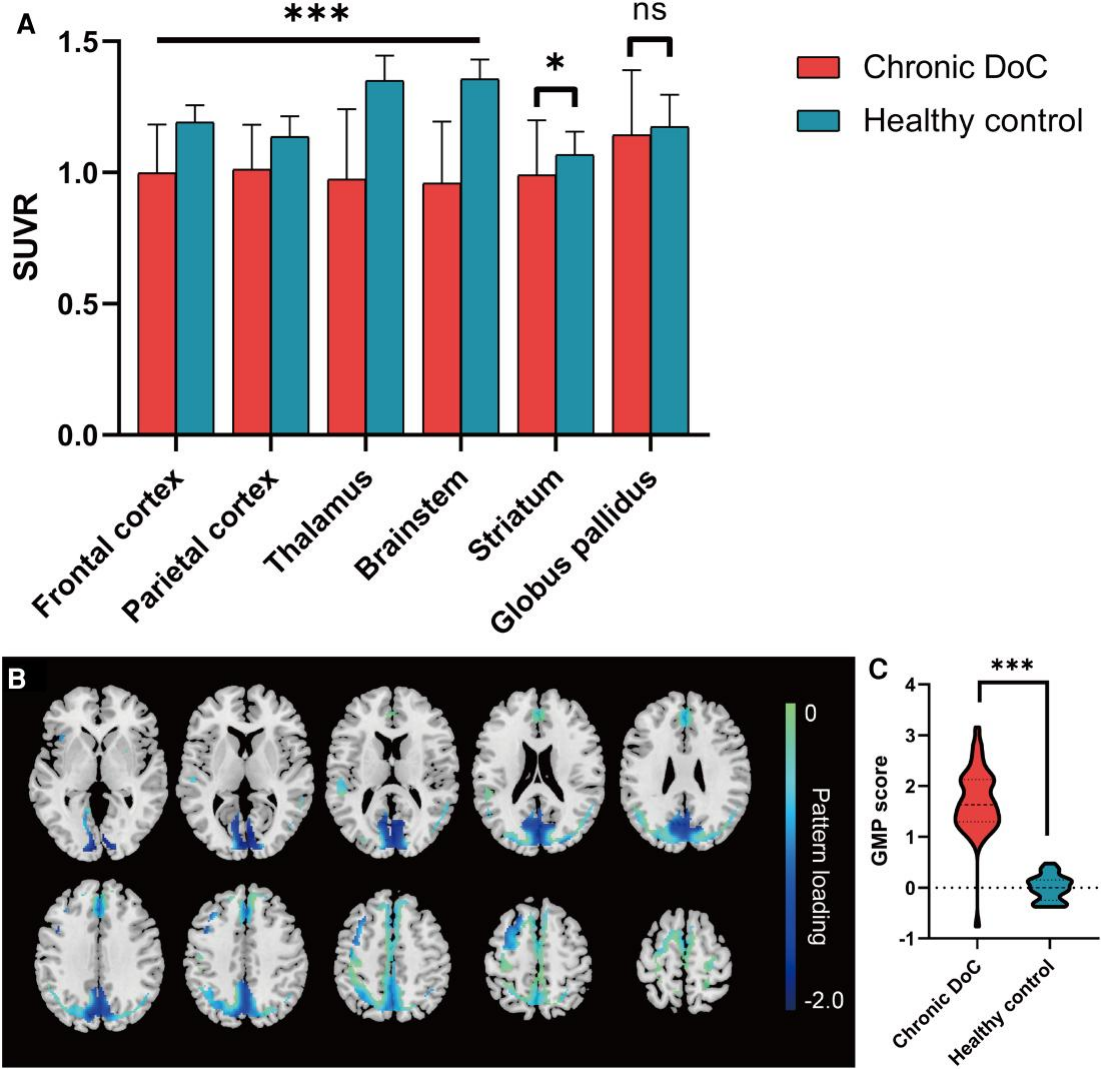
Glucose metabolism assessed by ^18^F-FDG PET in patients with chronic DoC and healthy controls. (**A**) Patients with chronic DoC (*n* = 41) showed significantly lower SUVRs in the frontal cortex, parietal cortex, thalamus, brainstem and striatum compared with healthy controls (*n* = 26), while the globus pallidus did not show a statistically significant difference. Group comparisons were performed using the Mann–Whitney U-test (frontal cortex: *U* = 586, *P* < 0.001; parietal cortex: *U* = 801, *P* < 0.001; thalamus: *U* = 349, *P* < 0.001; striatum: *U* = 1609, *P* = 0.017; globus pallidus: *U* = 1861, *P* = 0.215). (**B**) The GMP was identified from ^18^F-FDG PET data using SSM/PCA. Metabolic reductions were observed bilaterally in the frontal and parietal cortex. (**C**) Individual expression scores of the GMP distinguished patients with chronic DoC from healthy controls (*U* = 26, *P* < 0.001). DoC, disorders of consciousness; FDG, fluorodeoxyglucose; GMP, glucose metabolic pattern; SSM/PCA, scaled subprofile model/principal component analysis; SUVR, standardized uptake value ratio.

Compared to HCs, all mesocircuit-associated regions showed significantly lower SUVRs in the chronic DoC group, including the frontal cortex, parietal cortex, thalamus and brainstem (all *P* < 0.001). The difference in striatum was also statistically significant (*P* = 0.017), whereas the difference in the globus pallidus was not statistically significant (*P* > 0.05) (Fig. 4A).

**Figure 5:**
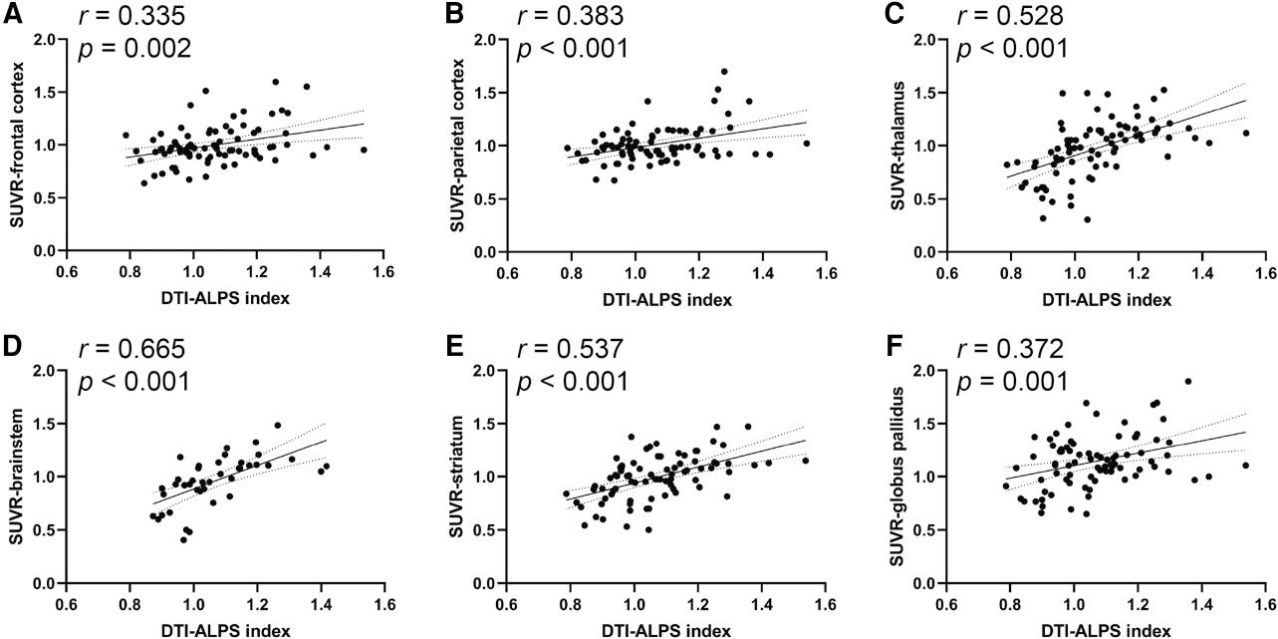
Correlations of the DTI-ALPS index with SUVR in patients with chronic DoC. Partial Pearson correlation analysis adjusting for age was performed to examine the associations between the DTI-ALPS index and SUVRs in patients with chronic DoC (*n* = 41). Significant correlations were observed for the frontal cortex (**A**), parietal cortex (**B**), thalamus (**C**), brainstem (**D**), striatum (**E**) and globus pallidus (**F**). Each dot represents a hemispheric measurement for bilateral regions and a subject-level measurement for the brainstem. DoC, disorders of consciousness; DTI-ALPS, diffusion tensor image analysis along the perivascular space; SUVR, standardized uptake value ratio.

In patients with chronic DoC, after adjusting for age, correlation analyses revealed moderate-to-strong correlations between the DTI-ALPS index and the SUVRs of mesocircuit-associated regions, including the frontal cortex (*r* = 0.335, *P* = 0.002), parietal cortex (*r* = 0.383, *P* < 0.001), thalamus (*r* = 0.528, *P* < 0.001), brainstem (*r* = 0.665, *P* < 0.001), striatum (*r* = 0.537, *P* < 0.001) and globus pallidus (*r* = 0.372, *P* = 0.001) (Fig. 5). All correlations remained statistically significant after false discovery rate correction for multiple comparisons.

**Figure 6:**
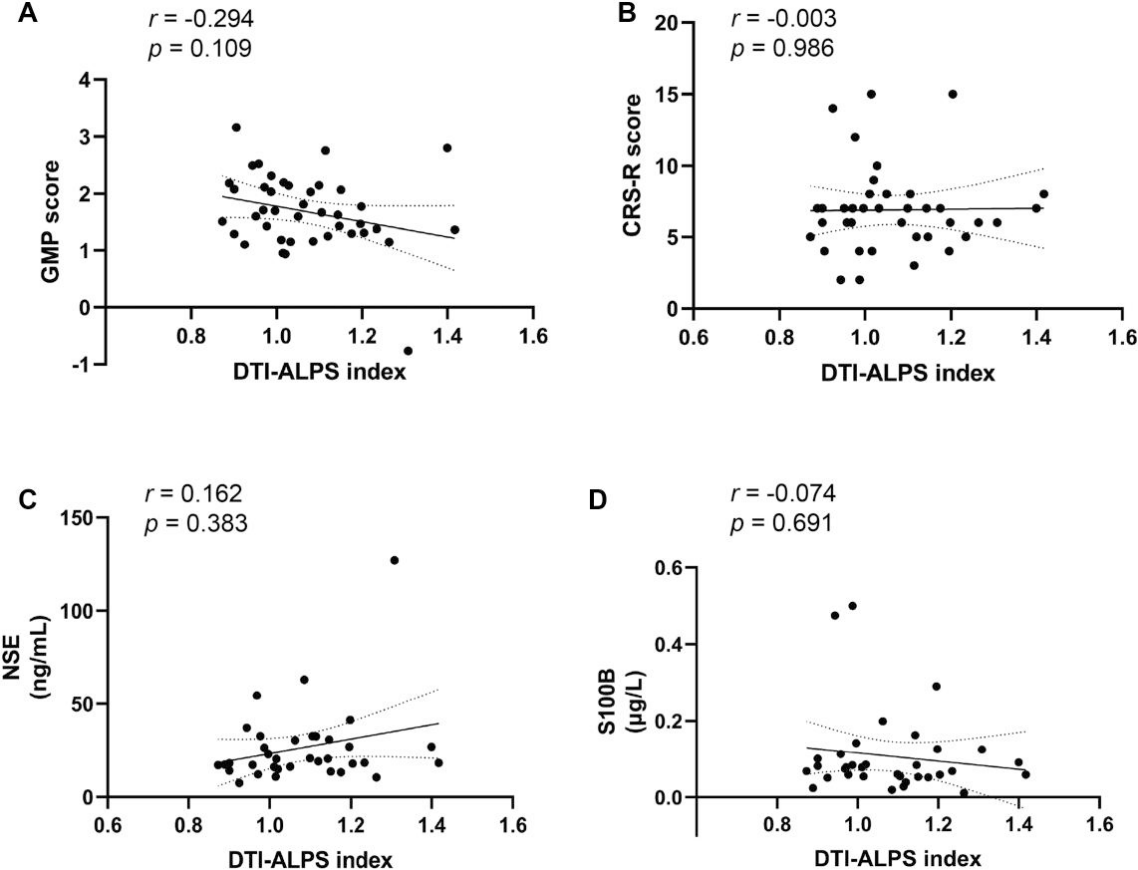
Correlations of the DTI-ALPS index with GMP score, CRS-R score and serum brain injury biomarker levels in patients with chronic DoC. Partial Pearson correlation analysis adjusting for age was performed to examine the associations between the DTI-ALPS index and the GMP score (**A**), CRS-R score (**B**), serum NSE level (**C**) and serum S100B level (**D**). No significant correlations were observed. The sample sizes were *n* = 41 for the GMP score and CRS-R score analyses, *n* = 36 for NSE and *n* = 34 for S100B. Each dot represents one individual patient. Abbreviations: CRS-R, Coma Recovery Scale-Revised; DoC, disorders of consciousness; DTI-ALPS, diffusion tensor image analysis along the perivascular space; GMP, glucose metabolic pattern; NSE, neuron-specific enolase; S100B, S100 calcium-binding protein B.

Compared to HCs, patients with chronic DoC exhibited widespread cortical hypometabolism, specifically in the frontal and parietal cortices (Fig. 4B). Patients with chronic DoC also exhibited significantly higher GMP scores (*P* < 0.001) (Fig. 4C). However, no significant correlation was found between the DTI-ALPS index and GMP scores in patients with chronic DoC, regardless of outliers (*P* > 0.05) (Fig. 6A).

**Figure 7:**
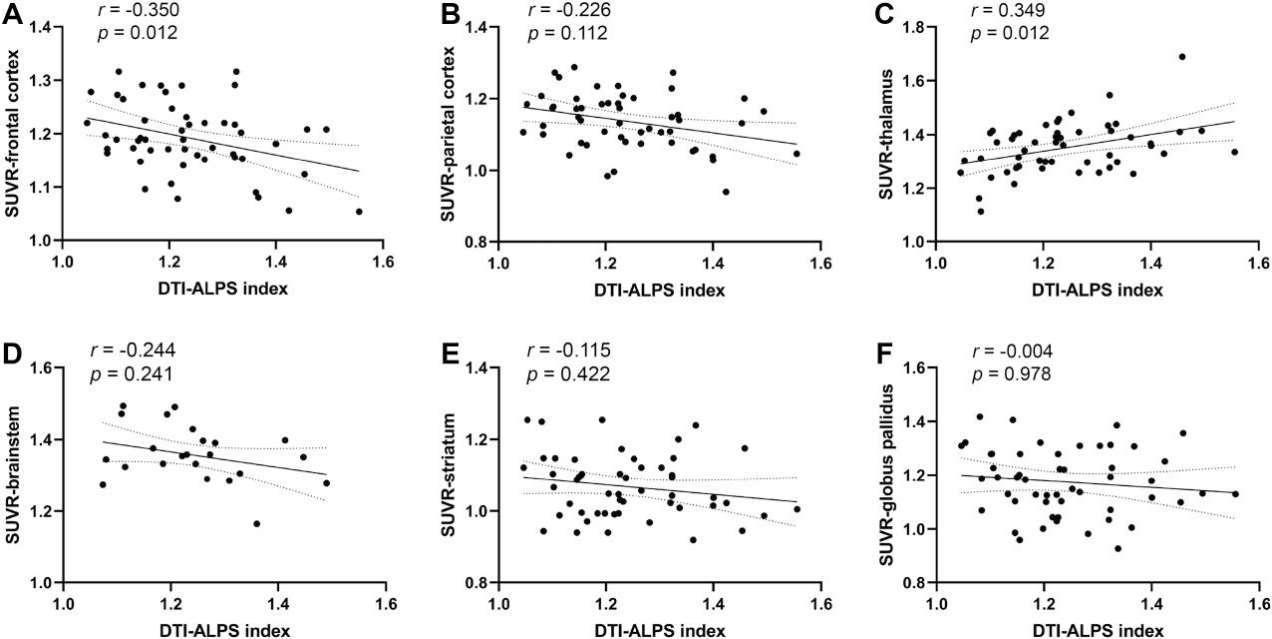
Correlations of the DTI-ALPS index with SUVR in healthy controls. Partial Pearson correlation analysis adjusting for age was performed to examine the associations between the DTI-ALPS index and SUVRs in healthy controls (*n* = 26). Significant correlations were observed for the frontal cortex (**A**) and thalamus (**C**), but not for the parietal cortex (**B**), brainstem (**D**), striatum (**E**), or globus pallidus (**F**). Each dot represents a hemispheric measurement for bilateral regions and a subject-level measurement for the brainstem. Abbreviations: DTI-ALPS, diffusion tensor image analysis along the perivascular space; SUVR, standardized uptake value ratio.

Among HCs, the DTI-ALPS index was moderately correlated with the SUVRs of the frontal cortex (*r* = −0.350, *P* = 0.012) and thalamus (*r* = 0.349, *P* = 0.012), but not with those of the parietal cortex, brainstem, striatum, or globus pallidus (all *P* > 0.05) (Fig. 7).

### Glymphatic function showed no significant association with either behavioural score or serological markers

Regardless of outlier removal, no significant correlation was found between the DTI-ALPS index and the CRS-R score after adjusting for age (*P* > 0.05) (Fig. 6B). Excluding the three PET-adjudicated patients yielded consistent results, indicating that the absence of association between DTI-ALPS and behavioural responsiveness was not driven by PET-assisted classification. Similarly, the DTI-ALPS index was not significantly correlated with serum NSE or S100B levels (both *P* > 0.05) (Fig. 6C, D).

### Ageing and regional cortical-subcortical hypometabolism were independently associated with glymphatic dysfunction

A multivariate linear regression analysis was conducted considering age and the SUVRs of the frontal cortex, parietal cortex, thalamus, brainstem, striatum and globus pallidus as independent variables. The results revealed that a greater age (*β* = −0.312, *P* < 0.001) and lower SUVR in the parietal cortex (*β* = 0.196, *P* = 0.030) and brainstem (*β* = 0.482, *P* < 0.001) were significantly associated with a lower DTI-ALPS index in patients with chronic DoC.

## Discussion

Our study used the noninvasive DTI-ALPS index to confirm that patients with chronic DoC have glymphatic dysfunction, and we found that the severity of this dysfunction (reflected by a lower DTI-ALPS index) was significantly associated with regional cerebral glucose hypometabolism. Furthermore, multivariate regression showed that older age and reduced glucose metabolism in the parietal cortex and brainstem independently predicted a lower DTI-ALPS index, suggesting that neural activity in these regions is a key physiological driver of CSF circulation in this population.

Recent evidence indicates that neural oscillations are a major driving force behind glymphatic perfusion, primarily by modulating vasomotion and ionic waves in the interstitial fluid.^15,21,22^ In this framework, reduced neuronal activity, reflected by diminished glucose metabolism, may attenuate vasomotion and weaken the ionic waves that help propel CSF along perivascular spaces. In patients with chronic DoC, the parietal cortex is one of the most consistently affected cortical regions and plays a central role in large-scale networks, such as the frontoparietal and default mode networks, that support conscious processing.^23^ Reduced glucose metabolism in the parietal cortex may therefore reflect diminished large-scale cortical activity, which could weaken the neural and vascular oscillations that help drive CSF movement along perivascular spaces. Meanwhile, the brainstem regulates autonomic and respiratory rhythms that influence vascular pulsatility and CSF dynamics, suggesting that hypometabolism in this region may further reduce the physiological forces that facilitate glymphatic circulation.^24^ Collectively, hypometabolism in the parietal cortex and brainstem could weaken the driving force, slowing CSF movement and impairing glymphatic clearance. This interpretation aligns with a previous BOLD-CSF coupling study in chronic DoC patients, which reported reduced coupling strength in the frontal and temporal lobes, suggesting a diminished capacity of these cortical regions to recruit CSF flow.^14^ Together, these findings support a neurovascular-glymphatic coupling model in which cortical and subcortical oscillations dynamically regulate CSF movement and waste clearance.

Beyond these oscillatory mechanisms, the structural impact of brain injury can also disrupt glymphatic pathways. The leading causes of chronic DoC, traumatic brain injury and hypoxic-ischaemic injury, trigger widespread neuronal loss, gliosis and neuroinflammation that impair glymphatic flow.^25-27^ Experimental studies demonstrate that glymphatic function can decrease by up to 60% after traumatic brain injury and remain impaired for weeks due to astrocytic swelling and loss of aquaporin-4 polarization at perivascular endfeet.^9^ This disorganization of perivascular astrocytes compromises fluid exchange between interstitial and perivascular compartments, leading to the accumulation of phosphorylated tau and axonal degeneration, which are closely linked to cognitive decline.^13^ Aging provides an additional challenge: in aged brains, aquaporin-4 channels lose their polarized localization at perivascular astrocytic endfeet, which undermines interstitial fluid clearance and leads to the accumulation of neurotoxic proteins.^28^ Consistently, our study identified age as an independent predictor of glymphatic impairment, supporting the contribution of ageing to glymphatic dysfunction.

Sleep is also an important modulator of glymphatic function because neural oscillations during sleep facilitate perivascular CSF influx.^15,29,30^ However, patients with chronic DoC often show severely disturbed sleep-wake cycles and typically lack normal sleep spindles or rapid eye movement sleep, particularly those diagnosed with UWS.^31^ Therefore, the combined effects of brain injury, ageing and sleep dysregulation may synergistically contribute to the reduced DTI-ALPS index observed in these patients. Future research should investigate whether interventions aimed at restoring aquaporin-4 polarization or promoting sleep integrity can enhance glymphatic clearance and potentially aid neurological recovery.

Although glymphatic dysfunction was significantly associated with regional glucose hypometabolism, its correlations with composite or systemic measures, such as GMP scores, CRS-R scores and serological biomarkers, were weak or nonsignificant. These findings suggest that the DTI-ALPS index primarily reflects regional neural-glymphatic coupling rather than global disease severity or behavioural responsiveness. This interpretation is consistent with the notion that recovery of consciousness depends on large-scale network reintegration, whereas glymphatic flow is a localized biophysical process.^32^ Several factors could also explain the lack of correlation with serological biomarkers. Glymphatic activity fluctuates over time, so the timing of blood collection may not have coincided with periods of high glymphatic activity. Moreover, clinical care practices (e.g. frequent vital sign monitoring that interrupts sleep) can disturb glymphatic function because it is strongly modulated by sleep.^30,33^ Pre-imaging blood collection could be an optimal time point to avoid alterations in glymphatic activity. Future longitudinal studies integrating imaging with peripheral biomarkers are warranted to determine whether glymphatic measures can complement clinical and molecular indices for prognosis evaluation.

In addition to clearing waste, the glymphatic system aids in distributing vital nutrients such as lipids and glucose.^34,35^ Glucose and its analogues, including ^18^F-FDG, travel through the perivascular space before being taken up by astrocytes and neurons.^35^ Glymphatic dysfunction may therefore reduce glucose availability in neural tissue, providing a mechanistic link between impaired CSF circulation and regional hypometabolism. Therefore, hypometabolism and glymphatic dysfunction may reinforce each other, forming a vicious cycle in patients with chronic DoC.

This study has several limitations. First, the retrospective single-centre design and modest sample size may limit the generalizability of our findings. Diagnostic classification relied primarily on repeated CRS-R assessments, and ^18^F-FDG PET was used to clarify classification in a small number of behaviourally indeterminate cases, which may introduce potential incorporation bias. Second, the retrospective design precludes causal inference between glymphatic dysfunction and regional cerebral glucose hypometabolism. Prospective longitudinal studies are therefore needed to determine the directionality of this association. Third, several methodological limitations should be considered. The DTI-ALPS index is an indirect marker of glymphatic function and may be influenced by structural alterations common in chronic DoC, such as white matter injury and ventricular enlargement. Although patients with major anatomical distortion were excluded, residual structural confounding cannot be completely excluded. In addition, the brainstem ROI was manually delineated without formal inter-rater reproducibility assessment, which may introduce measurement variability. Semi-quantitative ^18^F-FDG PET analysis relied on cerebellar SUVR normalization, and partial volume correction was not applied, both of which may influence PET quantification. Finally, sleep disturbances, which are common in chronic DoC and may modulate glymphatic activity, were not quantitatively assessed. Future multicenter studies with larger cohorts and multimodal imaging approaches are needed to further validate these findings.

## Conclusions

Glymphatic dysfunction in patients with chronic DoC correlates with reduced regional cerebral glucose metabolism, suggesting that reduced neural activity, particularly within the parietal cortex and brainstem, may weaken the force that drives CSF circulation and metabolic clearance.

## Supplementary Material

fcag235_Supplementary_Data

## Data Availability

The data that support the findings of this study are available from the corresponding author upon reasonable request. The analyses were performed using publicly available software tools, including the ScAnVP toolbox (https://feinsteinneuroscience.org/imaging-software/download-software) and the DTI-ALPS processing pipeline available at https://github.com/gbarisano/alps/.
