# Supplementary material for: Association between glymphatic dysfunction and glucose hypometabolism in chronic disorders of consciousness: a multimodal PET/MRI study
Source: Brain Commun. 2026 Jun 24;8(4):fcag235. doi: 10.1093/braincomms/fcag235 (PMC13326948; doi:10.1093/braincomms/fcag235)
Supplement: fcag235_Supplementary_Data [file fcag235_supplementary_data.docx]

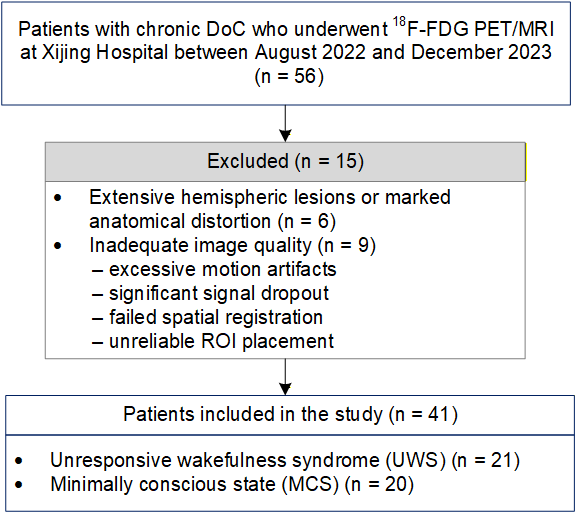


**Supplementary Fig.1 Flow diagram of patient selection.**

Flow diagram summarizing the patient selection process, including inclusion and exclusion criteria, for the chronic disorders of consciousness cohort included in this study (n = 41).
